# Multimodal interventional bronchoscopy for bronchial myelolipoma complicated with airway obstruction: a case report and literature review

**DOI:** 10.3389/fmed.2026.1936465

**Published:** 2026-08-26

**Authors:** Rongrong Zhou, Huaxu Yin, Ting Wang, Xing Chen, Jie Zhang, Xin Xiong, Bing Xue

**Affiliations:** Department of Respiratory and Critical Care Medicine, Chuiyangliu Hospital Affiliated to Tsinghua University, Beijing, China

**Keywords:** airway obstruction, bronchial myelolipoma, bronchoscopic interventional therapy, electrosurgical snare, laser ablation

## Abstract

Myelolipoma predominantly arises within the adrenal glands, while endobronchial myelolipoma is extremely rare and frequently leads to airway obstruction. We report a case of bronchial myelolipoma causing left lower lobe airway obstruction in a 62-year-old male who presented with cough and sputum. Chest computed tomography (CT) revealed a mixed-density lesion with calcification within the left lower lobe bronchial lumen, and the diagnosis was confirmed by bronchoscopic biopsy pathology. The patient underwent interventional minimally invasive interventional therapy under general anesthesia, employing flexible bronchoscopic electrosurgical snare resection combined with semiconductor laser ablation, which successfully removed the tumor and restored airway patency. We systematically reviewed the domestic and international literature and summarized all reported cases of bronchial myelolipoma (a total of 6 cases, including the present case), with a focus on the clinical decision-making basis for bronchoscopic interventional therapy versus surgical resection, and the technical considerations for selecting laser ablation over argon plasma coagulation (APC) and cryotherapy. This case demonstrates that electrosurgical snare combined with laser ablation is a safe and effective minimally invasive interventional treatment for airway obstruction caused by bronchial myelolipoma, providing a reference for the clinical diagnosis and management of this rare benign airway tumor.

## Introduction

Myelolipoma is a rare mesenchymal-derived benign tumor (1) characterized by the intermingling of mature adipose tissue and normal hematopoietic tissue, typically lacking endocrine function (2, 3). This disease predominantly occurs in the adrenal glands, with a prevalence of approximately 3–5% (1, 2); extra-adrenal myelolipoma is exceedingly rare, accounting for only 15% of all myelolipomas (3), and may involve the presacral region, retroperitoneum, liver, spleen, stomach, leptomeninges, and thoracic cavity (including the lung) (1). Intrathoracic myelolipoma accounts for approximately 3% of all myelolipoma cases and most commonly arises in the posterior mediastinum (2, 3). To date, approximately 21 cases of pulmonary myelolipoma have been reported worldwide, among which only 6 cases (including the present case) are purely bronchial in origin without involvement of the lung parenchyma (3–6).

Pulmonary myelolipoma is typically asymptomatic in early stages; tumor enlargement raises risks of secondary hemorrhage and compression of adjacent bronchi (1, 7). Traditionally, surgical resection has been the mainstay of treatment (2, 4), but it is associated with significant trauma and prolonged recovery. In recent years, with the rapid advancement of interventional pulmonology techniques, bronchoscopic minimally invasive therapy has been increasingly employed to relieve both benign and malignant airway obstruction (8–10).

We report a case of bronchial myelolipoma causing airway obstruction that was successfully treated by bronchoscopic electrosurgical snare resection combined with semiconductor laser ablation. We also systematically reviewed the domestic and international literature and performed a pooled analysis of all published cases of bronchial myelolipoma to provide a reference for the clinical diagnosis and management of this disease entity.

## Case report

**Figure 1:**
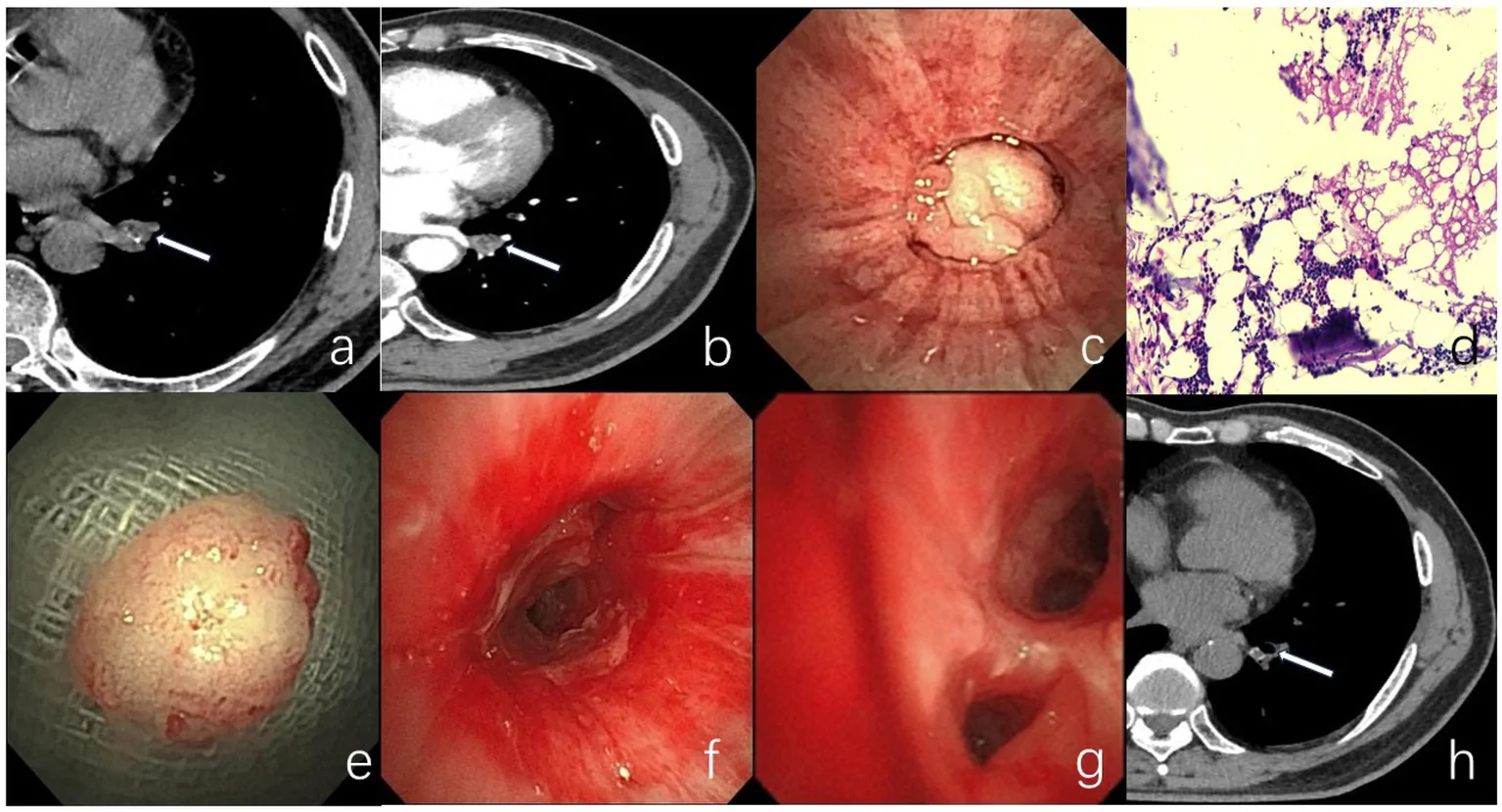
**(a)** Chest CT showing a mixed-density lesion with calcification within the orifice of the left lower lobe basal segment; **(b)** Contrast-enhanced CT showing enhancement of portions of the soft tissue within the lesion, without evidence of bronchial wall infiltration; **(c)** Bronchoscopic view showing a neoplasm completely obstructing the orifice of the left lower lobe basal segment, with a smooth and lobulated surface; **(d)** Pathological microscopy: Mature adipose tissue and hematopoietic tissue with ossification; **(e)** Partial tumor resection using an electrosurgical snare, yielding a specimen approximately 1.0 × 0.8 cm; **(f)** Patency of the left lower lobe basal segment orifice after electrosurgical snare resection and laser ablation; **(g)** Patent distal bronchial orifices of the left lower lobe basal segment following bronchoscopic interventional therapy; **(h)** Follow-up chest CT at 2 months post-intervention showing patency of the left lower lobe basal segment bronchus.

A 62-year-old male was admitted in April 2026 with 8 days of cough and sputum, without fever or hemoptysis. His past medical history included hypertension, coronary heart disease, and chronic obstructive pulmonary disease (COPD). He had a 40-year smoking history of approximately 20 cigarettes per day and had not quit. Physical examination on admission revealed sparse moist crackles in the left lower lung field. Laboratory investigations—including arterial blood gas analysis, complete blood count, liver and renal function tests, cardiac enzymes, tumor markers, coagulation profile, autoantibodies, immunoglobulins, thyroid function, procalcitonin, and erythrocyte sedimentation rate—were all unremarkable. Urinary tract ultrasonography showed no obvious abnormalities in the bilateral kidneys or ureters. Pulmonary function tests revealed moderate obstructive ventilation disorder, with a negative bronchodilator response test. Chest CT revealed bronchiectasis in the left lower lobe, with a mixed-density lesion accompanied by calcification within the lumen of the left lower lobe basal segment orifice and part of the distal bronchus (Figure 1a). Contrast-enhanced chest CT showed a mixed-density opacity within the left lower lobe basal segment orifice and part of the distal bronchus, with enhancement of portions of the soft tissue but no evidence of bronchial wall infiltration (Figure 1b).

On hospital day 3, bronchoscopic exploration was performed. Bronchoscopy revealed a neoplasm completely obstructing the lumen of the left lower lobe basal segment, with a smooth, lobulated surface (Figure 1c). Biopsy forceps were used to grasp the tumor, which was firm in consistency and difficult to avulse; therefore, an electrosurgical snare was used to resect a portion of the tumor for pathological examination, and brush cytology was performed at the lesion site, with minimal intraoperative bleeding. Brush cytology revealed no malignant cells. Pathological examination of the specimen showed a grayish-yellow, firm, nodular tissue; microscopy revealed mature adipose tissue and hematopoietic tissue with ossification (Figure 1d), consistent with the diagnosis of myelolipoma. The tumor completely obstructed the orifice of the left lower lobe basal segment and extended into part of the distal bronchus, posing a risk of recurrent infection and atelectasis, thus constituting a clear indication for therapeutic intervention.

On hospital day 6, bronchoscopic interventional therapy was performed under general anesthesia: (1) Anesthesia and bronchoscopy approach: The patient was placed in the supine position. General anesthesia was induced intravenously (propofol + sufentanil + rocuronium), and a rigid bronchoscope was inserted and connected to high-frequency jet ventilation. A flexible bronchoscope (Aohua Therapeutic Flexible Bronchoscope VBC-T300, working channel 3.0 mm) was introduced through the rigid bronchoscope via the oral route, sequentially passing through the vocal cords, trachea, and carina, entering the left main bronchus to reach the orifice of the left lower lobe basal segment. (2) Equipment parameters: ① Electrosurgical snare (Olympus SD-7P) with a high-frequency electrosurgical unit (ERBE VIO 200D) set as follows: cutting mode (ENDO CUT Q, EFFECT 3), peak voltage 770 Vp; coagulation mode (FORCED COAG), power 40 W. ② Semiconductor laser (JXmedex LaserPro D980), wavelength 980 nm, non-contact mode, power set at 10 W, duration 0.5–2.0 s. Intraoperative fractional inspired oxygen (FiO₂) was controlled <40% to eliminate airway ignition risk, and SpO₂ was maintained above 90%. (3) Sequence of surgical procedures: ① Biopsy forceps (Olympus FB-56D-1) were first used to attempt debulking of the tumor; however, due to the firm consistency of the tumor, the forceps had difficulty grasping the tissue. ② The electrosurgical snare was then substituted. The snare loop was placed around the base of the tumor, the wire was slowly tightened, and the lesion was lifted upward to separate it from the bronchial wall. The tumor was resected using the cutting mode (yellow foot pedal, 6-s automatic pulsed intermittent mode), yielding a specimen approximately 1.0 × 0.8 cm in size (Figure 1e). ③ The tumor was found to have a broad base, and the residual basal tissue required thorough ablation. Semiconductor laser was therefore combined: the laser fiber was advanced through the working channel, with the fiber tip positioned at least 1 cm from the bronchoscope tip and approximately 5–10 mm from the target tissue. The laser beam was emitted parallel to the airway wall (angle >40°), and continuous output mode at 10 W power was used to progressively vaporize and ablate the residual basal tissue until the lumen regained patency (Figure 1f). ④ A large amount of purulent secretion extravasated from the distal basal segment, which was irrigated with 37 °C normal saline in aliquots (5–10 mL each) and thoroughly aspirated. (4) Criteria for assessing complete airway recanalization: Intraoperative assessment of airway recanalization was based on the following composite criteria: ① no residual mass or obstruction was visible under direct bronchoscopic visualization; ② the 4.9 mm outer-diameter bronchoscope could be smoothly passed through the treated segment into the distal bronchus; ③ the distal bronchial orifices were visible and patent; ④ secretions from the distal bronchus could be smoothly aspirated. The present case met all of the above criteria, and complete recanalization was determined. Intraoperative blood loss was approximately 5 mL, with no serious complications such as perforation or airway fire. Follow-up bronchoscopy was performed 4 days postoperatively: a small amount of necrotic tissue was found adherent to the lumen of the left lower lobe basal segment, which was repeatedly grasped and removed with biopsy forceps; purulent secretions from the distal airway were aspirated and irrigated; and the distal bronchial orifices were observed to be patent (Figure 1g). At 2 months postoperatively (June 2026), follow-up chest CT demonstrated patency of the previously obstructed left lower lobe basal segment bronchus, with resolution of the previously observed mixed-density lesion (Figure 1h). The patient’s symptoms of cough and sputum had completely resolved.

## Discussion

Myelolipoma was first described in 1905 and formally named in 1929 (11). The disease predominantly affects individuals aged 45–81 years, with a male predominance (1, 3, 7). Bronchial myelolipoma is exceedingly rare, with only 6 cases reported globally to date (Table 1). Its pathogenesis remains incompletely understood and may involve hypotheses including embryonic mesenchymal cell differentiation, hematogenous embolization and colonization of bone marrow hematopoietic cells, reticuloendothelial cell metaplasia, abnormal proliferation of hematopoietic stem cells, and chromosomal gene mutations (3, 11, 12). Pathological biopsy is the gold standard for definitive diagnosis; microscopy reveals mature adipose tissue and hematopoietic tissue, occasionally with osseous tissue (3, 7, 12). Differential diagnoses include extramedullary hematopoiesis, lipoma, hamartoma, ectopic bone marrow, and teratoma (1, 3, 7, 12).

**Table 1:** Summary of published cases of bronchial myelolipoma.

No.	Author(s)	Year	Tumor location	Treatment modality	Follow-up duration	Notes
1	Liu LY (6)	2012	Left main bronchus	Bronchoscopic resection (details not described)	Not mentioned	With osseous and chondroid metaplasia; 63-year-old male
2	Chung HS (2)	2019	Right intermediate bronchus	Rigid bronchoscopic resection combined with semiconductor laser ablation of the base	12 months (no recurrence)	Granulation tissue formation at 3 months, resolved by 12 months; 38-year-old male
3	Ji JL (3)	2023	Multiple bilateral bronchi	Cryobiopsy with liquid nitrogen + APC hemostasis only	Not mentioned	First case of multiple endobronchial lesions; 71-year-old male; with COPD and diabetes
4	Yan L (4)	2024	Left main bronchus	Thoracoscopic lobectomy combined with bronchoplasty	Not mentioned	With fibroepithelial polyp, focal amyloidosis; rare combination
5	Su HY (5)	2024	Right intermediate bronchus	Electrosurgical snare + CO₂ cryotherapy	6 years (no recurrence)	72-year-old male
6	Present case	2026	Left lower lobe basal segment	Electrosurgical snare resection + semiconductor laser ablation of the base	2 months (no recurrence)	Broad base; with ossification; 62-year-old male

Search databases: PubMed, Web of Science, CNKI, Wanfang Data; search cutoff: June 2026. Only purely endobronchial myelolipoma cases without parenchymal involvement were included.

Currently, there are no unified treatment guidelines for pulmonary myelolipoma. It is generally considered that asymptomatic patients with small lesions (< 4 cm) require no treatment and can be managed with regular follow-up; patients with clinical symptoms, large lesions (> 7 cm), or progressive growth are typically advised to undergo surgical resection due to the risk of secondary hemorrhage or compression of adjacent bronchi, and postoperative complications and recurrence are uncommon (12, 13). Endobronchial tumors, however, are prone to causing luminal narrowing or obstruction, leading to cough, sputum, hemoptysis, recurrent pneumonia, and atelectasis; resection is generally advocated to prevent and treat these complications (2, 8). Although traditional surgical resection remains the classic therapeutic approach, it is associated with significant trauma and prolonged postoperative recovery. In recent years, bronchoscopic interventional techniques have developed rapidly and are gradually replacing traditional surgery as the first-line treatment for benign and malignant central airway obstruction. These techniques include thermal ablation (laser, electrocautery, APC, photodynamic therapy), cryotherapy, balloon dilation, and airway stenting, which are frequently used in combination to enhance therapeutic efficacy (9, 10).

To systematically review the current status of diagnosis and treatment of bronchial myelolipoma, we summarized all published relevant cases (Table 1). To date, a total of 6 cases (including the present case) of myelolipoma purely originating from the bronchial lumen without involvement of the lung parenchyma have been reported worldwide, of which one case was complicated by fibroepithelial polyp (4) and one case involved multiple bilateral bronchial lesions (3). Regarding treatment modalities, 4 cases underwent bronchoscopic treatment: one case did not describe the specific treatment method (6), and 3 cases [Su (5), Chung (2), and the present case] received bronchoscopic interventional therapy (electrosurgical snare/laser); one case underwent surgical resection (4), and one case underwent cryobiopsy with APC for hemostasis only (3). In terms of follow-up, one case had no recurrence at 12 months (2), one case had no recurrence at 6 years (5), and the present case was followed up for only 2 months. The present case is the second reported case in China of bronchial myelolipoma treated by bronchoscopic intervention and the first case to employ the combined “electrosurgical snare plus semiconductor laser ablation” technique for this disease. Its distinctive feature is that the tumor had a broad base and was accompanied by ossification, which increased the technical difficulty of the interventional procedure.

The decision to pursue bronchoscopic intervention rather than surgical resection in this case was based on the following considerations: (1) Tumor nature and airway obstruction characteristics: The pathological diagnosis of benign myelolipoma had been confirmed. CT and bronchoscopy indicated that the tumor completely obstructed the orifice of the left lower lobe basal segment and extended into part of the distal bronchus, with the lesion was confined to the bronchial lumen, without invasion of the full-thickness bronchial wall or pulmonary parenchyma. According to the criteria proposed by Guibert et al. (14) for bronchoscopic treatment of benign endobronchial tumors—strictly limited to intraluminal polypoid tumors (base <15 mm^2^) without submucosal invasion—although the present case had a broad base, the lesion was confined to the lumen, and contrast-enhanced CT revealed no signs of extraluminal invasion, thus meeting the anatomical conditions for bronchoscopic resection. Furthermore, the British Thoracic Society (BTS) guideline for advanced diagnostic and therapeutic flexible bronchoscopy in adults (15) explicitly recommends that electrocautery/electrocoagulation can be used for curative treatment of benign airway diseases, including benign tumors. (2) Patient baseline status and surgical risk assessment: The patient was 62 years old with underlying comorbidities including hypertension, coronary heart disease, and COPD, and a 40-year smoking history. Pulmonary function testing demonstrated moderate obstructive ventilation disorder. Traditional surgical resection (lobectomy or sleeve resection) is associated with significant trauma and prolonged postoperative recovery; for this patient with cardiopulmonary comorbidities, the surgical risk was substantially increased. Scarlata et al. (16) reported their experience with rigid bronchoscopic resection of 57 benign tracheobronchial tumors over a 12-year period, achieving a complete eradication rate of 63.1% in a single procedure, with only 12.3% requiring conversion to surgery due to multiple recurrences, and no major complications, demonstrating that endoscopic treatment can serve as a first-line therapy for benign airway tumors, particularly for elderly patients at high surgical risk. (3) Treatment goals: The primary goal was to relieve airway obstruction and eliminate the risk of recurrent infection and atelectasis. Bronchoscopic intervention can precisely target intraluminal lesions, achieving an equivalent degree of obstruction relief as surgical resection while maximally preserving lung parenchyma and pulmonary function. (4) Minimally invasive advantages: Bronchoscopic intervention offers the advantages of minimal trauma, rapid recovery, and shortened hospital stay. (5) Support from previous similar case experience: Chung et al. (2) first reported bronchoscopic interventional treatment of solitary bronchial myelolipoma in 2019, employing rigid bronchoscopic resection combined with semiconductor laser ablation of the base, with no recurrence at 12-month follow-up; Su et al. (5) used electrosurgical snare combined with carbon dioxide cryotherapy to resect solitary bronchial myelolipoma, with no recurrence at 6-year follow-up. These experiences demonstrate that bronchoscopic interventional treatment of bronchial myelolipoma is safe and effective, providing direct evidence-based support for the selection of the procedural approach in this case.

However, it must be emphasized that bronchoscopic minimally invasive treatment is applicable only to a strictly selected subset of patients—namely those with intraluminal tumors, no full-thickness airway wall invasion or extraluminal involvement, and who can tolerate general anesthesia for bronchoscopic procedures. For cases with extensive airway wall invasion, parenchymal involvement requiring lobectomy, extraluminal compression, recurrence or malignant transformation after multiple endoscopic treatments, or inability to establish a definitive pathological diagnosis, surgical resection remains an irreplaceable treatment option.

In this case, after partial tumor resection with the electrosurgical snare, the tumor was found to have a broad base, and the residual basal tissue carried a high risk of bleeding and perforation, necessitating further ablation to ensure complete eradication and prevent recurrence. In selecting the ablation modality, semiconductor laser was preferred over APC or cryotherapy based on the following clinical considerations: (1) Ablation depth and curability: Laser (e.g., Nd: YAG or semiconductor laser) can achieve penetration depths of several millimeters or deeper, enabling effective vaporization and resection of broad-based residual tumor tissue and reducing the risk of recurrence (2, 17); in contrast, APC has a relatively shallow tissue penetration depth (typically 2–3 mm) and is primarily suitable for coagulation, hemostasis, and superficial ablation of superficial lesions (17), with limited capability for thorough clearance of broad-based residual tissue. Shepherd et al. (17) noted that due to its shallow penetration depth, APC has limited efficacy when used alone for larger lesions and is better suited as an adjunctive hemostatic modality or for superficial lesion management. In the present case, the tumor was firm in consistency and broad-based, making the deep cutting advantage of laser more prominent. (2) Hemostatic efficacy and safety: In this case, only minimal bleeding occurred after combined electrosurgical snare resection. Laser can coagulate blood vessels while vaporizing tissue, effectively controlling wound surface bleeding, and offers high operational precision, enabling precise ablation of the base while avoiding damage to the surrounding normal bronchial wall. (3) Limitations of cryotherapy: Cryotherapy (including cryoprobes and cryobiopsy) offers advantages in obtaining large tissue specimens, but its ablation effect is delayed, making it unsuitable for intraoperative immediate hemostasis and real-time airway recanalization (17); Ji et al. (3) employed cryobiopsy combined with APC for the treatment of multiple bronchial myelolipomas, and multiple masses partially persisted postoperatively, suggesting that cryotherapy has limitations in achieving complete lesion clearance. Considering these factors comprehensively, laser ablation was selected for the broad-based residual tissue in this case, balancing the clinical needs for ablation depth, hemostatic efficacy, and immediate airway recanalization.

The combined electrosurgical snare and laser ablation approach employed in this case offers the following advantages: (1) the electrosurgical snare enables rapid resection of the main tumor body, procurement of intact pathological specimens, and prompt restoration of the obstructed lumen; (2) laser ablation provides precise vaporization of broad-based residual tissue, reducing the risk of recurrence and compensating for the limitations of a single instrument; (3) the combination balances debulking efficiency with basal tissue management. This is consistent with the multimodal combined strategy concept reported by Dalar et al. (18)—initially using laser or electrocautery for rapid debulking, followed by management of the base and hemostasis—yielding superior efficacy compared with any single technique. This approach also has the following limitations: (1) the electrosurgical snare carries a risk of residual tissue when resecting broad-based, firm tumors, necessitating laser rescue ablation; (2) laser ablation may cause thermal damage to the bronchial wall, with long-term risks of granulation tissue formation and scar stenosis—the case reported by Chung et al. (2) developed granulation tissue formation at 3 months postoperatively, requiring removal with biopsy forceps, underscoring the need for close postoperative follow-up; (3) this combined technique requires the operator to have extensive experience in interventional pulmonology, with precise control of laser power and irradiation time to avoid serious complications such as perforation and massive hemorrhage. It is recommended that interventional pulmonologists performing such combined procedures should thoroughly evaluate the tumor base width, consistency, and blood supply preoperatively, prepare hemostatic instruments (e.g., APC, epinephrine) intraoperatively, and conduct regular postoperative bronchoscopic follow-up to detect granulation hyperplasia or recurrence at an early stage.

Currently, there are no disease-specific diagnostic and treatment guidelines for bronchial myelolipoma. The treatment protocol in this study can be correlated with the following authoritative guidelines. First, the American College of Chest Physicians (ACCP) Clinical Practice Guideline for the Management of Central Airway Obstruction (10) recommends that for patients with symptomatic central airway obstruction, therapeutic bronchoscopy should be selected as an adjunct to systemic therapy based on multidisciplinary team assessment and shared decision-making, with multimodal interventional modalities (rigid bronchoscopy, ablation, dilation, stenting, etc.) used in combination as appropriate. The treatment strategy in this case is highly consistent with the “multimodal combined intervention” concept recommended by this guideline. Second, Insler et al. (8) noted in their clinicopathological review of benign endobronchial tumors that bronchoscopic intervention is an effective modality for relieving airway obstruction caused by benign endobronchial tumors, and its safety has been validated across multiple benign tumor types (e.g., hamartoma, chondroma, leiomyoma). As a benign mesenchymal tumor, the interventional treatment strategy for bronchial myelolipoma can be guided by the management principles for these benign endobronchial tumors. Furthermore, Rosell and Stratakos (9) emphasized in their review on therapeutic bronchoscopy that thermal ablation techniques (laser, electrocautery, APC, etc.) frequently require combination use to enhance therapeutic efficacy; the strategy of combining electrosurgical snare with laser in this case is consistent with this consensus. In summary, in the absence of disease-specific guidelines, the treatment protocol in this study is consistent with the principles of existing authoritative guidelines for benign airway tumors and therapeutic bronchoscopy, and may serve as a reference for clinical decision-making in similar rare cases.

Based on the present case and prior literature, the following clinical recommendations are proposed for interventional pulmonologists: ① Regarding preoperative assessment: It is recommended to perform contrast-enhanced chest CT to evaluate the tumor extent, base width, and airway wall invasion. When available, radial probe endobronchial ultrasound (RP-EBUS) should be combined to assess cartilaginous layer invasion (sensitivity 86%, specificity 100%) (14); ② Regarding procedural selection strategy: For intraluminal, pedunculated bronchial myelolipomas, electrosurgical snare resection may be the first-line approach; for broad-based, firm, or ossified tumors, electrosurgical snare for rapid debulking combined with laser ablation of the base is recommended; for multiple lesions or superficial residual tissue, combination with APC or cryotherapy may be considered (3, 18); ③ Regarding complication prevention and management: Postoperative attention should be paid to granulation tissue formation [Chung et al. (2) reported granulation tissue formation at 3 months postoperatively]. Regular postoperative bronchoscopic follow-up is recommended, and intraoperative control of laser power and FiO₂ (< 40%) is essential to prevent perforation and airway fire (19, 20); ④ Regarding follow-up recommendations: Regular bronchoscopy and chest CT examinations are recommended at 3, 6, and 12 months postoperatively, followed by annual follow-up for at least 2–3 years.

This study has the following limitations: (1) Short follow-up duration. The postoperative follow-up period in this case was only 2 months, substantially shorter than that of previous similar cases [Chung et al. (2) reported no recurrence at 12-month follow-up; Su et al. (5) reported no recurrence at 6-year follow-up]. A 2-month follow-up period is insufficient to evaluate long-term efficacy and recurrence risk, particularly regarding the fate of residual basal tissue after laser ablation of broad-based tumors, which requires longer-term observation. (2) This is a single-case report with a small sample size and lack of a control group, precluding statistical analysis; the generalizability of the conclusions is therefore limited. (3) RP-EBUS was not used for preoperative assessment of cartilaginous layer invasion; reliance on contrast-enhanced CT and intraoperative visual assessment may have underestimated the extent of wall invasion. (4) Bronchoscopic minimally invasive treatment is applicable only to a strictly selected patient population (intraluminal type, no full-thickness wall invasion, no extraluminal involvement) and cannot be generalized to all cases of bronchial myelolipoma.

## Conclusion

In conclusion, bronchial myelolipoma is an exceedingly rare endobronchial benign tumor that is prone to clinical misdiagnosis. The core of diagnosis and management lies in enhancing imaging recognition capabilities, establishing a definitive diagnosis based on pathology, and prioritizing bronchoscopic minimally invasive interventional therapy for appropriately selected patients. In this case, electrosurgical snare combined with semiconductor laser ablation successfully resected the lesion and eliminated the risk of recurrent infection and atelectasis caused by obstruction, further validating the safety and efficacy of interventional pulmonology techniques in this disease. Through literature review and the case summary table, the uniqueness and positioning of this case among all previously reported cases were clarified—as the 6th case of bronchial myelolipoma worldwide, the 2nd case treated by bronchoscopic intervention in China, and the first case treated with the combined “electrosurgical snare plus semiconductor laser ablation” technique.

However, it must be noted that the follow-up period of this study was only 2 months, significantly shorter than that of previous similar cases, and is insufficient to evaluate long-term efficacy and recurrence risk. Prolonged follow-up is needed to ascertain long-term prognosis. Furthermore, bronchoscopic minimally invasive treatment is applicable only to a strictly selected patient population and cannot entirely replace surgical resection. As a single-case report, this study requires corroboration from large-sample prospective studies to confirm long-term safety and efficacy. Moreover, this disease entity necessitates multidisciplinary collaboration between interventional pulmonology and thoracic surgery to achieve individualized precision diagnosis and treatment.

## Patient perspective

The patient expressed satisfaction with the treatment process and outcome. Preoperatively, the symptoms of cough and sputum severely affected daily life; following bronchoscopic interventional therapy, these symptoms completely resolved. The patient acknowledged that the minimally invasive treatment avoided the need for thoracotomy and expressed willingness to comply with long-term follow-up.

## Data Availability

The original contributions presented in the study are included in the article/supplementary material, further inquiries can be directed to the corresponding authors.
